# 4-[(*E*)-2,6-Dichloro­benzyl­ideneamino]-3-{1-[4-(2-methyl­prop­yl)phen­yl]eth­yl}-1*H*-1,2,4-triazole-5(4*H*)-thione

**DOI:** 10.1107/S1600536808021272

**Published:** 2008-07-16

**Authors:** Hoong-Kun Fun, Suchada Chantrapromma, K. V. Sujith, P. S. Patil, B. Kalluraya, A. Muralidharan, S. M. Dharmaprakash

**Affiliations:** a X-ray Crystallography Unit, School of Physics, Universiti Sains Malaysia, 11800 USM, Penang, Malaysia; b Crystal Materials Research Unit, Department of Chemistry, Faculty of Science, Prince of Songkla University, Hat-Yai, Songkhla 90112, Thailand; c Department of Studies in Chemistry, Mangalore University, Mangalagangotri, Mangalore 574 199, India; d Department of Studies in Physics, Mangalore University, Mangalagangotri, Mangalore 574 199, India; e Department of Chemistry, Nehru Arts & Science College, Kanhangad, Kerala 671 328, India

## Abstract

In the title Schiff base compound, C_21_H_22_Cl_2_N_4_S, the triazole ring makes dihedral angles of 2.15 (11) and 87.48 (11)° with the 2,6-dichloro­phenyl and methyl­propyl­phenyl rings, respectively. Weak intra­molecular C—H⋯S and C—H⋯Cl inter­actions generate *S*(6) and *S*(5) ring motifs, respectively. In the crystal structure, centrosymmetrically related mol­ecules are linked into dimers by N—H⋯S hydrogen bonds. These dimers are arranged into sheets parallel to the *ab* plane and are stacked along the *c* axis. C—H⋯π inter­actions involving the methyl­propyl­phenyl ring and π–π inter­actions involving the dichloro­phenyl ring [centroid–centroid distance = 3.5865 (3) Å] are also observed.

## Related literature

For related literature on hydrogen-bond motifs, see: Bernstein *et al.* (1995 ▶). For bond-length data, see: Allen *et al.* (1987 ▶). For related structures, see: Fun *et al.* (2008*a* ▶,*b* ▶). For background to the activities and applications of 1,2,4-triazole derivatives, see: Almasirad *et al.* (2004 ▶); Al-Soud *et al.* (2003 ▶); Amir & Shikha (2004 ▶); Holla *et al.* (2003 ▶); Kawashima *et al.* (1987 ▶); Palaska *et al.* (2002 ▶); Walczak *et al.* (2004 ▶); Zitouni *et al.* (2005 ▶).
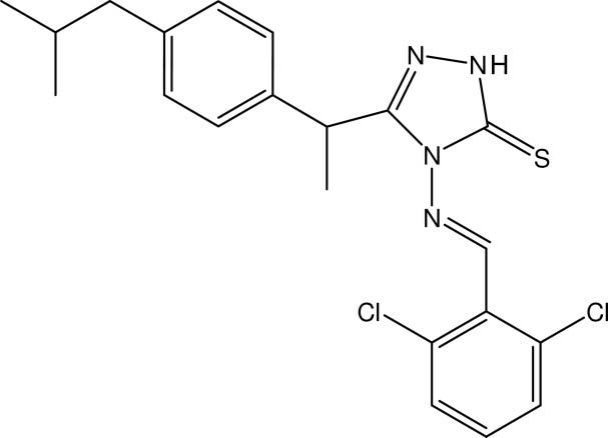


## Experimental

### 

#### Crystal data

C_21_H_22_Cl_2_N_4_S
*M* *_r_* = 433.40
Triclinic, 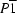

*a* = 8.6190 (2) Å
*b* = 9.4441 (2) Å
*c* = 14.4244 (4) Å
α = 104.669 (2)°
β = 95.492 (2)°
γ = 110.418 (1)°
*V* = 1042.33 (5) Å^3^
*Z* = 2
Mo *K*α radiation
μ = 0.43 mm^−1^
*T* = 100.0 (1) K
0.29 × 0.20 × 0.16 mm

#### Data collection

Bruker SMART APEXII CCD area-detector diffractometer
Absorption correction: multi-scan (*SADABS*; Bruker, 2005 ▶) *T*_min_ = 0.887, *T*_max_ = 0.934
19865 measured reflections
6032 independent reflections
4139 reflections with *I* > 2σ(*I*)
*R*_int_ = 0.050

#### Refinement

*R*[*F* ^2^ > 2σ(*F* ^2^)] = 0.046
*wR*(*F* ^2^) = 0.136
*S* = 1.00
6032 reflections
256 parameters
H-atom parameters constrained
Δρ_max_ = 0.59 e Å^−3^
Δρ_min_ = −0.47 e Å^−3^

### 

Data collection: *APEX2* (Bruker, 2005 ▶); cell refinement: *APEX2*; data reduction: *SAINT* (Bruker, 2005 ▶); program(s) used to solve structure: *SHELXTL* (Sheldrick, 2008 ▶); program(s) used to refine structure: *SHELXTL*; molecular graphics: *SHELXTL*; software used to prepare material for publication: *SHELXTL* and *PLATON* (Spek, 2003 ▶).

## Supplementary Material

Crystal structure: contains datablocks global, I. DOI: 10.1107/S1600536808021272/ci2627sup1.cif

Structure factors: contains datablocks I. DOI: 10.1107/S1600536808021272/ci2627Isup2.hkl

Additional supplementary materials:  crystallographic information; 3D view; checkCIF report

## Figures and Tables

**Table 1:** Hydrogen-bond geometry (Å, °) *Cg*1 is the centroid of the C1–C6 ring.

*D*—H⋯*A*	*D*—H	H⋯*A*	*D*⋯*A*	*D*—H⋯*A*
N2—H2*A*⋯S1^i^	0.86	2.44	3.2849 (19)	169
C10—H10*A*⋯Cl2	0.93	2.62	2.978 (2)	104
C10—H10*A*⋯S1	0.93	2.52	3.2066 (19)	131
C15—H15*A*⋯*Cg*1^ii^	0.93	2.94	3.793 (3)	154

Symmetry codes: (i) 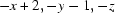; (ii) 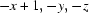.

**Fig. 1.**
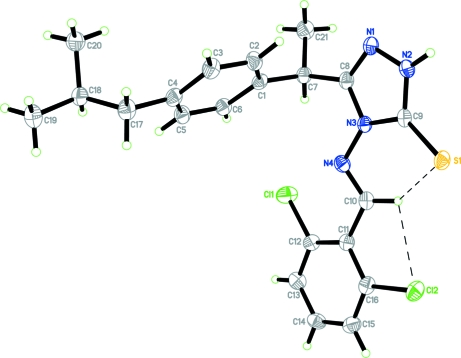
The structure of the title compound, showing 50% probability displacement ellipsoids and the atom-numbering scheme. Weak C—H···S and C—H···Cl hydrogen bonds are shown as dashed lines.

**Fig. 2.**
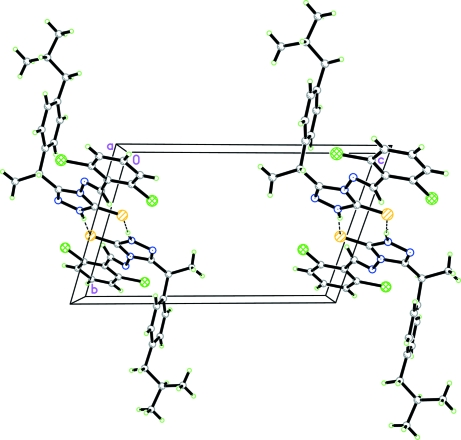
Part of the crystal packing of the title compound, viewed along the a axis, showing hydrogen-bonded (dashed lines) dimers.

**Fig. 3.**
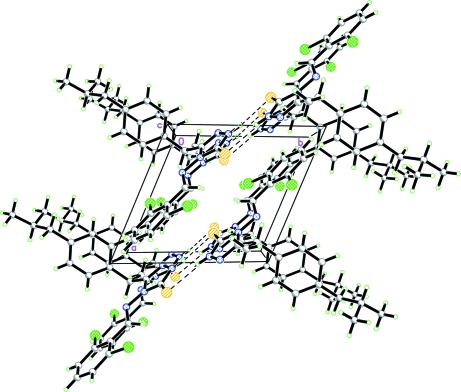
The packing diagram of the title compound, viewed along the c axis, showing stacking of the molecular sheets. Hydrogen bonds are shown as dashed lines.

## supplementary crystallographic information

### Comment

1,2,4-Triazoles and their derivatives represent an overwhelming and rapid developing field in modern heterocyclic chemistry. A degree of respectability has been bestowed for 1,2,4-triazole derivatives due to their antibacterial, antifungal (Zitouni *et al.*, 2005), antitubercular (Walczak *et al.*, 2004), anticancer (Holla *et al.*, 2003), antitumor (Al-Soud *et al.*, 2003), anticonvulsant (Almasirad *et al.*, 2004), antiinflammatory, and analgesic properties (Amir & Shikha, 2004). Certain 1,2,4-triazoles also find applications in the preparation of photographic plates, polymers, and as analytical agents (Kawashima *et al.*, 1987). Similarly, ibuprofen belongs to the class of Non-Steroidal Anti-Inflammatory Drugs (NSAIDs) with antipyretic, anti-inflammatory and analgesic properties (Palaska *et al.*, 2002). Our earlier studies involved synthesis of heterocyclic compounds which contain in their structures both the ibuprofen and 1,2,4-triazole fragments (Fun *et al.*, 2008*a*,*b*). In this connection and in continuation of our interest in the synthesis of chemically and biologically important heterocycles, we report here the crystal structure of a substituted 1,2,4-triazole Schiff base carrying only the ibuprofen moiety.

In the title Schiff base compound (Fig. 1), the 1,2,4 triazole ring (C8-C9/N1-N3) is planar with a maximum deviation of 0.009 (2) Å for atom N3. The 1,2,4 triazole ring is co-planar with the 2,6-dichlorophenyl (C11-C16) ring [dihedral angle = 2.15 (11)°] but is almost perpendicular to the methylpropylphenyl (C1-C6) ring with a dihedral angle of 87.48 (11)°. The methylidene amino linkage (N4/C10) is slightly twisted from the mean plane of the 1,2,4 triazole ring as indicated by the torsion angle C9–N3–N4–C10 of 26.4 (3)°. Weak C—H···S and C—H···Cl intramolecular interactions generate S(6) and S(5) ring motifs, respectively (Bernstein *et al.*, 1995). The bond distances and angles have normal values (Allen *et al.*, 1987) and are comparable with closely related structures (Fun *et al.*, 2008*a*,*b*).

In the crystal structure, centrosymmetrically related molecules are linked into dimers (Fig. 2) by N—H···S hydrogen bonds (Table 1). These dimers are arranged into sheets parallel to the *ab* plane and these sheets are stacked along the *c* axis (Fig. 3). In addition, the crystal structure is stabilized by C—H···π interactions (Table 1) involving the C1-C6 ring (centroid Cg1) and π-π interaction involving the C11-C16 ring (centroid Cg2) [*Cg*_2_···*Cg*_2_^ii^ = 3.5865 (3) Å, symmetry code: (ii) 1 - *x*, -*y*, -*z*].

### Experimental

The title compound was obtained by refluxing 4-amino-5-[1-(4-isobutylphenyl)ethyl]-4*H*-1,2,4-triazole-3-thiol (0.01 mol) and 2,6-dichlorobenzaldehyde (0.01 mol) in ethanol (50 ml) with the addition of 3 drops of concentrated sulfuric acid for 3 h. The solid product obtained was collected by filtration, washed with ethanol and dried. Colourless single crystals suitable for X-ray analysis were obtained from a acetone-*N*,*N*-dimethylformamide (DMF) (1:3 *v*/*v*) solution by slow evaporation (yield 53%; m.p. 438–440 K)

### Refinement

All H atoms were placed in calculated positions (N-H = 0.86 Å and C-H = 0.93-0.98 Å) and refined using a riding model, with U_iso_(H) = 1.2U_eq_(C,N) and 1.5U_eq_(C_methyl_). A rotating group model was used for the methyl groups.

### Crystal data

**Table:**

C_21_H_22_Cl_2_N_4_S	*Z* = 2
*M**_r_* = 433.40	*F*_000_ = 452
Triclinic, *P*1	*D*_x_ = 1.381 Mg m^−^^3^
Hall symbol: -P 1	Melting point = 438–440 K
*a* = 8.6190 (2) Å	Mo *K*α radiation λ = 0.71073 Å
*b* = 9.4441 (2) Å	Cell parameters from 6032 reflections
*c* = 14.4244 (4) Å	θ = 1.5–30.0º
α = 104.669 (2)º	µ = 0.43 mm^−^^1^
β = 95.492 (2)º	*T* = 100.0 (1) K
γ = 110.418 (1)º	Block, colourless
*V* = 1042.33 (5) Å^3^	0.29 × 0.20 × 0.16 mm

### Data collection

**Table:**

Bruker SMART APEXII CCD area-detector diffractometer	6032 independent reflections
Radiation source: fine-focus sealed tube	4139 reflections with *I* > 2σ(*I*)
Monochromator: graphite	*R*_int_ = 0.050
Detector resolution: 8.33 pixels mm^-1^	θ_max_ = 30.0º
*T* = 100.0(1) K	θ_min_ = 1.5º
ω scans	*h* = −12→12
Absorption correction: multi-scan(SADABS; Bruker, 2005)	*k* = −13→13
*T*_min_ = 0.887, *T*_max_ = 0.935	*l* = −20→19
19865 measured reflections	

### Refinement

**Table:**

Refinement on *F*^2^	Secondary atom site location: difference Fourier map
Least-squares matrix: full	Hydrogen site location: inferred from neighbouring sites
*R*[*F*^2^ > 2σ(*F*^2^)] = 0.046	H-atom parameters constrained
*wR*(*F*^2^) = 0.136	*w* = 1/[σ^2^(*F*_o_^2^) + (0.0746*P*)^2^] where *P* = (*F*_o_^2^ + 2*F*_c_^2^)/3
*S* = 1.00	(Δ/σ)_max_ = 0.001
6032 reflections	Δρ_max_ = 0.59 e Å^−^^3^
256 parameters	Δρ_min_ = −0.47 e Å^−^^3^
Primary atom site location: structure-invariant direct methods	Extinction correction: none

### Special details

**Table:**

Experimental. The low-temperature data was collected with the Oxford Cyrosystem Cobra low-temperature attachment.
Geometry. All e.s.d.'s (except the e.s.d. in the dihedral angle between two l.s. planes) are estimated using the full covariance matrix. The cell e.s.d.'s are taken into account individually in the estimation of e.s.d.'s in distances, angles and torsion angles; correlations between e.s.d.'s in cell parameters are only used when they are defined by crystal symmetry. An approximate (isotropic) treatment of cell e.s.d.'s is used for estimating e.s.d.'s involving l.s. planes.
Refinement. Refinement of *F*^2^ against ALL reflections. The weighted *R*-factor *wR* and goodness of fit *S* are based on *F*^2^, conventional *R*-factors *R* are based on *F*, with *F* set to zero for negative *F*^2^. The threshold expression of *F*^2^ > σ(*F*^2^) is used only for calculating *R*-factors(gt) *etc*. and is not relevant to the choice of reflections for refinement. *R*-factors based on *F*^2^ are statistically about twice as large as those based on *F*, and *R*- factors based on ALL data will be even larger.

### Fractional atomic coordinates and isotropic or equivalent isotropic displacement parameters (Å^2^)

**Table:**

	*x*	*y*	*z*	*U*_iso_*/*U*_eq_	
S1	0.79027 (6)	−0.43005 (6)	−0.06227 (3)	0.02335 (13)	
Cl1	0.43164 (7)	−0.07928 (7)	0.21127 (4)	0.03523 (15)	
Cl2	0.41695 (7)	−0.33808 (6)	−0.17537 (4)	0.03136 (14)	
N1	0.9611 (2)	−0.3270 (2)	0.21761 (12)	0.0244 (4)	
N2	0.9465 (2)	−0.3961 (2)	0.11923 (12)	0.0240 (4)	
H2A	1.0037	−0.4511	0.0971	0.029*	
N3	0.7738 (2)	−0.28053 (18)	0.12730 (11)	0.0192 (3)	
N4	0.6602 (2)	−0.20834 (19)	0.11584 (12)	0.0229 (4)	
C1	0.9409 (2)	0.0211 (2)	0.32320 (13)	0.0218 (4)	
C2	1.1052 (3)	0.0657 (2)	0.30759 (15)	0.0259 (4)	
H2B	1.1515	−0.0105	0.2915	0.031*	
C3	1.2013 (3)	0.2223 (2)	0.31568 (15)	0.0266 (4)	
H3A	1.3109	0.2492	0.3046	0.032*	
C4	1.1366 (3)	0.3403 (2)	0.34015 (14)	0.0244 (4)	
C5	0.9747 (3)	0.2957 (2)	0.35952 (14)	0.0262 (4)	
H5A	0.9301	0.3726	0.3786	0.031*	
C6	0.8779 (3)	0.1389 (2)	0.35107 (14)	0.0234 (4)	
H6A	0.7696	0.1125	0.3642	0.028*	
C7	0.8319 (3)	−0.1523 (2)	0.31079 (14)	0.0225 (4)	
H7A	0.7131	−0.1651	0.3013	0.027*	
C8	0.8563 (2)	−0.2560 (2)	0.22068 (14)	0.0217 (4)	
C9	0.8351 (2)	−0.3702 (2)	0.06064 (14)	0.0206 (4)	
C10	0.5498 (2)	−0.2695 (2)	0.03633 (14)	0.0223 (4)	
H10A	0.5495	−0.3578	−0.0105	0.027*	
C11	0.4228 (2)	−0.2036 (2)	0.01691 (14)	0.0215 (4)	
C12	0.3619 (3)	−0.1177 (2)	0.08757 (15)	0.0239 (4)	
C13	0.2388 (3)	−0.0628 (2)	0.06263 (16)	0.0270 (4)	
H13A	0.2011	−0.0054	0.1112	0.032*	
C14	0.1729 (3)	−0.0938 (2)	−0.03479 (16)	0.0278 (5)	
H14A	0.0906	−0.0570	−0.0516	0.033*	
C15	0.2278 (3)	−0.1792 (3)	−0.10761 (16)	0.0284 (5)	
H15A	0.1829	−0.2003	−0.1732	0.034*	
C16	0.3504 (3)	−0.2325 (2)	−0.08126 (14)	0.0246 (4)	
C17	1.2359 (3)	0.5077 (2)	0.34117 (15)	0.0281 (5)	
H17A	1.1787	0.5286	0.2881	0.034*	
H17B	1.3462	0.5147	0.3281	0.034*	
C18	1.2612 (3)	0.6380 (2)	0.43631 (15)	0.0248 (4)	
H18A	1.1498	0.6274	0.4512	0.030*	
C19	1.3448 (3)	0.8002 (2)	0.42311 (17)	0.0331 (5)	
H19A	1.3584	0.8813	0.4826	0.050*	
H19B	1.2752	0.8098	0.3709	0.050*	
H19C	1.4535	0.8121	0.4075	0.050*	
C20	1.3655 (3)	0.6216 (3)	0.52176 (15)	0.0307 (5)	
H20A	1.3751	0.7017	0.5808	0.046*	
H20B	1.4763	0.6345	0.5092	0.046*	
H20C	1.3109	0.5185	0.5289	0.046*	
C21	0.8713 (3)	−0.2028 (3)	0.39995 (15)	0.0315 (5)	
H21A	0.8010	−0.3124	0.3882	0.047*	
H21B	0.8496	−0.1387	0.4564	0.047*	
H21C	0.9880	−0.1890	0.4113	0.047*	

### Atomic displacement parameters (Å^2^)

**Table:**

	*U*^11^	*U*^22^	*U*^33^	*U*^12^	*U*^13^	*U*^23^
S1	0.0258 (3)	0.0241 (2)	0.0194 (2)	0.0112 (2)	0.00672 (19)	0.00244 (18)
Cl1	0.0389 (3)	0.0551 (4)	0.0221 (3)	0.0310 (3)	0.0104 (2)	0.0093 (2)
Cl2	0.0362 (3)	0.0338 (3)	0.0213 (3)	0.0135 (2)	0.0063 (2)	0.0036 (2)
N1	0.0296 (9)	0.0241 (8)	0.0190 (8)	0.0132 (7)	0.0055 (7)	0.0016 (7)
N2	0.0272 (9)	0.0240 (8)	0.0228 (8)	0.0152 (7)	0.0061 (7)	0.0030 (7)
N3	0.0210 (8)	0.0184 (7)	0.0182 (8)	0.0081 (6)	0.0061 (6)	0.0043 (6)
N4	0.0241 (8)	0.0230 (8)	0.0247 (9)	0.0120 (7)	0.0080 (7)	0.0072 (7)
C1	0.0245 (10)	0.0248 (10)	0.0144 (9)	0.0095 (8)	0.0034 (7)	0.0036 (7)
C2	0.0280 (10)	0.0235 (10)	0.0270 (11)	0.0134 (8)	0.0080 (8)	0.0033 (8)
C3	0.0245 (10)	0.0280 (10)	0.0245 (10)	0.0090 (8)	0.0086 (8)	0.0037 (8)
C4	0.0299 (10)	0.0252 (10)	0.0159 (9)	0.0108 (8)	0.0050 (8)	0.0023 (8)
C5	0.0293 (10)	0.0250 (10)	0.0242 (10)	0.0140 (8)	0.0062 (8)	0.0020 (8)
C6	0.0245 (10)	0.0261 (10)	0.0205 (9)	0.0124 (8)	0.0072 (8)	0.0037 (8)
C7	0.0251 (10)	0.0229 (9)	0.0195 (9)	0.0103 (8)	0.0064 (8)	0.0042 (7)
C8	0.0249 (10)	0.0194 (9)	0.0199 (9)	0.0075 (8)	0.0069 (7)	0.0051 (7)
C9	0.0227 (9)	0.0165 (8)	0.0212 (9)	0.0067 (7)	0.0071 (7)	0.0039 (7)
C10	0.0216 (9)	0.0203 (9)	0.0237 (10)	0.0066 (8)	0.0081 (8)	0.0054 (7)
C11	0.0192 (9)	0.0203 (9)	0.0235 (10)	0.0049 (7)	0.0062 (8)	0.0072 (7)
C12	0.0228 (10)	0.0270 (10)	0.0223 (10)	0.0093 (8)	0.0068 (8)	0.0082 (8)
C13	0.0225 (10)	0.0300 (11)	0.0323 (11)	0.0125 (8)	0.0106 (8)	0.0113 (9)
C14	0.0219 (10)	0.0300 (11)	0.0356 (12)	0.0107 (9)	0.0062 (9)	0.0158 (9)
C15	0.0274 (10)	0.0296 (11)	0.0255 (11)	0.0074 (9)	0.0028 (8)	0.0103 (8)
C16	0.0246 (10)	0.0244 (10)	0.0226 (10)	0.0065 (8)	0.0079 (8)	0.0064 (8)
C17	0.0349 (12)	0.0259 (10)	0.0224 (10)	0.0104 (9)	0.0104 (9)	0.0060 (8)
C18	0.0267 (10)	0.0220 (10)	0.0241 (10)	0.0086 (8)	0.0062 (8)	0.0053 (8)
C19	0.0390 (13)	0.0245 (10)	0.0335 (12)	0.0102 (10)	0.0105 (10)	0.0070 (9)
C20	0.0353 (12)	0.0306 (11)	0.0264 (11)	0.0164 (9)	0.0049 (9)	0.0046 (9)
C21	0.0416 (13)	0.0319 (11)	0.0234 (11)	0.0159 (10)	0.0103 (9)	0.0088 (9)

### Geometric parameters (Å, °)

**Table:**

S1—C9	1.6797 (19)	C10—C11	1.472 (3)
Cl1—C12	1.730 (2)	C10—H10A	0.93
Cl2—C16	1.740 (2)	C11—C12	1.398 (3)
N1—C8	1.297 (2)	C11—C16	1.409 (3)
N1—N2	1.377 (2)	C12—C13	1.389 (3)
N2—C9	1.345 (2)	C13—C14	1.381 (3)
N2—H2A	0.86	C13—H13A	0.93
N3—C8	1.384 (2)	C14—C15	1.382 (3)
N3—C9	1.385 (2)	C14—H14A	0.93
N3—N4	1.394 (2)	C15—C16	1.381 (3)
N4—C10	1.275 (2)	C15—H15A	0.93
C1—C2	1.388 (3)	C17—C18	1.531 (3)
C1—C6	1.390 (3)	C17—H17A	0.97
C1—C7	1.530 (3)	C17—H17B	0.97
C2—C3	1.388 (3)	C18—C19	1.519 (3)
C2—H2B	0.93	C18—C20	1.527 (3)
C3—C4	1.396 (3)	C18—H18A	0.98
C3—H3A	0.93	C19—H19A	0.96
C4—C5	1.388 (3)	C19—H19B	0.96
C4—C17	1.508 (3)	C19—H19C	0.96
C5—C6	1.389 (3)	C20—H20A	0.96
C5—H5A	0.93	C20—H20B	0.96
C6—H6A	0.93	C20—H20C	0.96
C7—C8	1.498 (3)	C21—H21A	0.96
C7—C21	1.529 (3)	C21—H21B	0.96
C7—H7A	0.98	C21—H21C	0.96
			
C8—N1—N2	104.17 (15)	C13—C12—Cl1	116.33 (16)
C9—N2—N1	114.24 (15)	C11—C12—Cl1	121.52 (16)
C9—N2—H2A	122.9	C14—C13—C12	119.57 (19)
N1—N2—H2A	122.9	C14—C13—H13A	120.2
C8—N3—C9	108.43 (15)	C12—C13—H13A	120.2
C8—N3—N4	119.01 (15)	C13—C14—C15	120.7 (2)
C9—N3—N4	132.41 (16)	C13—C14—H14A	119.6
C10—N4—N3	117.04 (16)	C15—C14—H14A	119.6
C2—C1—C6	117.99 (19)	C16—C15—C14	118.81 (19)
C2—C1—C7	121.30 (17)	C16—C15—H15A	120.6
C6—C1—C7	120.71 (18)	C14—C15—H15A	120.6
C1—C2—C3	121.00 (18)	C15—C16—C11	122.96 (19)
C1—C2—H2B	119.5	C15—C16—Cl2	117.26 (16)
C3—C2—H2B	119.5	C11—C16—Cl2	119.79 (16)
C2—C3—C4	121.3 (2)	C4—C17—C18	115.60 (17)
C2—C3—H3A	119.4	C4—C17—H17A	108.4
C4—C3—H3A	119.4	C18—C17—H17A	108.4
C5—C4—C3	117.32 (19)	C4—C17—H17B	108.4
C5—C4—C17	121.41 (18)	C18—C17—H17B	108.4
C3—C4—C17	121.22 (19)	H17A—C17—H17B	107.4
C4—C5—C6	121.51 (18)	C19—C18—C20	110.68 (17)
C4—C5—H5A	119.2	C19—C18—C17	109.89 (18)
C6—C5—H5A	119.2	C20—C18—C17	111.28 (17)
C5—C6—C1	120.83 (19)	C19—C18—H18A	108.3
C5—C6—H6A	119.6	C20—C18—H18A	108.3
C1—C6—H6A	119.6	C17—C18—H18A	108.3
C8—C7—C21	110.54 (16)	C18—C19—H19A	109.5
C8—C7—C1	108.88 (16)	C18—C19—H19B	109.5
C21—C7—C1	112.70 (16)	H19A—C19—H19B	109.5
C8—C7—H7A	108.2	C18—C19—H19C	109.5
C21—C7—H7A	108.2	H19A—C19—H19C	109.5
C1—C7—H7A	108.2	H19B—C19—H19C	109.5
N1—C8—N3	110.81 (16)	C18—C20—H20A	109.5
N1—C8—C7	126.05 (18)	C18—C20—H20B	109.5
N3—C8—C7	123.04 (17)	H20A—C20—H20B	109.5
N2—C9—N3	102.32 (15)	C18—C20—H20C	109.5
N2—C9—S1	127.33 (15)	H20A—C20—H20C	109.5
N3—C9—S1	130.33 (15)	H20B—C20—H20C	109.5
N4—C10—C11	121.60 (18)	C7—C21—H21A	109.5
N4—C10—H10A	119.2	C7—C21—H21B	109.5
C11—C10—H10A	119.2	H21A—C21—H21B	109.5
C12—C11—C16	115.84 (18)	C7—C21—H21C	109.5
C12—C11—C10	125.92 (18)	H21A—C21—H21C	109.5
C16—C11—C10	118.21 (17)	H21B—C21—H21C	109.5
C13—C12—C11	122.11 (19)		
			
C8—N1—N2—C9	0.0 (2)	N1—N2—C9—N3	−1.0 (2)
C8—N3—N4—C10	−158.60 (18)	N1—N2—C9—S1	177.52 (14)
C9—N3—N4—C10	26.4 (3)	C8—N3—C9—N2	1.52 (19)
C6—C1—C2—C3	−2.6 (3)	N4—N3—C9—N2	176.87 (18)
C7—C1—C2—C3	177.75 (18)	C8—N3—C9—S1	−176.89 (16)
C1—C2—C3—C4	0.3 (3)	N4—N3—C9—S1	−1.5 (3)
C2—C3—C4—C5	2.3 (3)	N3—N4—C10—C11	179.51 (17)
C2—C3—C4—C17	−175.13 (19)	N4—C10—C11—C12	−26.3 (3)
C3—C4—C5—C6	−2.6 (3)	N4—C10—C11—C16	155.8 (2)
C17—C4—C5—C6	174.83 (19)	C16—C11—C12—C13	−0.6 (3)
C4—C5—C6—C1	0.3 (3)	C10—C11—C12—C13	−178.53 (19)
C2—C1—C6—C5	2.3 (3)	C16—C11—C12—Cl1	177.38 (15)
C7—C1—C6—C5	−178.04 (18)	C10—C11—C12—Cl1	−0.6 (3)
C2—C1—C7—C8	−42.3 (2)	C11—C12—C13—C14	0.3 (3)
C6—C1—C7—C8	138.03 (19)	Cl1—C12—C13—C14	−177.75 (16)
C2—C1—C7—C21	80.7 (2)	C12—C13—C14—C15	0.1 (3)
C6—C1—C7—C21	−98.9 (2)	C13—C14—C15—C16	−0.2 (3)
N2—N1—C8—N3	1.0 (2)	C14—C15—C16—C11	−0.1 (3)
N2—N1—C8—C7	−175.37 (18)	C14—C15—C16—Cl2	−179.50 (16)
C9—N3—C8—N1	−1.7 (2)	C12—C11—C16—C15	0.5 (3)
N4—N3—C8—N1	−177.76 (17)	C10—C11—C16—C15	178.60 (19)
C9—N3—C8—C7	174.84 (17)	C12—C11—C16—Cl2	179.88 (15)
N4—N3—C8—C7	−1.2 (3)	C10—C11—C16—Cl2	−2.0 (2)
C21—C7—C8—N1	−29.1 (3)	C5—C4—C17—C18	56.3 (3)
C1—C7—C8—N1	95.2 (2)	C3—C4—C17—C18	−126.4 (2)
C21—C7—C8—N3	154.92 (18)	C4—C17—C18—C19	−173.51 (18)
C1—C7—C8—N3	−80.8 (2)	C4—C17—C18—C20	63.5 (2)

### Hydrogen-bond geometry (Å, °)

**Table:**

*D*—H···*A*	*D*—H	H···*A*	*D*···*A*	*D*—H···*A*
N2—H2A···S1^i^	0.86	2.44	3.2849 (19)	169
C10—H10A···Cl2	0.93	2.62	2.978 (2)	104
C10—H10A···S1	0.93	2.52	3.2066 (19)	131
C15—H15A···Cg1^ii^	0.93	2.94	3.793 (3)	154

Symmetry codes: (i) −*x*+2, −*y*−1, −*z*;  (ii) −*x*+1, −*y*, −*z*.
